# Identification of Enzymes-specific Protein Domain Based on DDE, and Convolutional Neural Network

**DOI:** 10.3389/fgene.2021.759384

**Published:** 2021-11-30

**Authors:** Rahu Sikander, Yuping Wang, Ali Ghulam, Xianjuan Wu

**Affiliations:** ^1^ School of Computer Science and Technology, Xidian University, Xi’an, China; ^2^ Computerization and Network Section, Sindh Agriculture University, Tando Jam, Pakistan

**Keywords:** enzyme, function, sequence, protein, machine learing

## Abstract

Predicting the protein sequence information of enzymes and non-enzymes is an important but a very challenging task. Existing methods use protein geometric structures only or protein sequences alone to predict enzymatic functions. Thus, their prediction results are unsatisfactory. In this paper, we propose a novel approach for predicting the amino acid sequences of enzymes and non-enzymes via Convolutional Neural Network (CNN). In CNN, the roles of enzymes are predicted from multiple sides of biological information, including information on sequences and structures. We propose the use of two-dimensional data via 2DCNN to predict the proteins of enzymes and non-enzymes by using the same fivefold cross-validation function. We also use an independent dataset to test the performance of our model, and the results demonstrate that we are able to solve the overfitting problem. We used the CNN model proposed herein to demonstrate the superiority of our model for classifying an entire set of filters, such as 32, 64, and 128 parameters, with the fivefold validation test set as the independent classification. Via the Dipeptide Deviation from Expected Mean (DDE) matrix, mutation information is extracted from amino acid sequences and structural information with the distance and angle of amino acids is conveyed. The derived feature maps are then encoded in DDE exploitation*.* The independent datasets are then compared with other two methods, namely, GRU and XGBOOST. All analyses were conducted using 32, 64 and 128 filters on our proposed CNN method. The cross-validation datasets achieved an accuracy score of 0.8762%, whereas the accuracy of independent datasets was 0.7621%. Additional variables were derived on the basis of ROC AUC with fivefold cross-validation was achieved score is 0.95%. The performance of our model and that of other models in terms of sensitivity (0.9028%) and specificity (0.8497%) was compared. The overall accuracy of our model was 0.9133% compared with 0.8310% for the other model.

## Introduction

Enzymes are at the core of biological processes because their reactions are vital biological activities. Enzymes catalyze and spread to all parts of organisms and are involved in all biochemical reactions. Therefore, no metabolism can be reasonably assumed to occur if enzymes are absent. Enzymes are important because living organisms cannot survive with-out enzymatic reactions and biotechnological industries cannot generate products without enzymatic support. A key goal of contemporary molecular biology is to understand the laws governing the three-dimensional protein structures of amino acid sequences (R. Apweiler, et al., 2004). Experimentally and theoretically, proteins can be considered as enzymes and non-enzymes. A critical problem in the post-genome era is the prediction and classification of the primary sequences of proteins (Altschul et al., 1997). The number of proteins available in public and private databases is growing exponentially, and new methods for understanding and classifying them must be established. The enormous task of determining the functions of proteins has led to the development of more advanced methods for automated protein classification for any protein entry (Altschul et al., 1997; Altschul et al., 1997a; Jones, 1999). An automated measurement method for evaluating protein function based on its sequence is one of the key problems in bioinformatics. This task is a time-consuming, and a faster classification method is obviously needed. Structural protein groups are closely associated with the composition of amino acids. They have marked the beginning of using algorithms aimed solely at predicting amino acid compositions of structural protein types (A. Krizhevsky, et al., 2012). Aside from amino acid compositions, prediction accuracy is high when the sequence order is considered along the primary protein structure (N. Srivastava,et al., 2014).

The prediction of protein functions is becoming increasingly relevant as it enables the properties of novel proteins to be calculated. Protein function is determined by the arrangement of proteins and the sequence of amino acids. For example, the protein structure, that is, the protein’s 3D structure, is a very strong indicator of protein function (Illergård et al., 2009). Given that protein structure is related to amino acid sequences, more details can be directly derived from amino acid sequences (Dehzangi et al., 2017). For example, sequence homology is vital in predicting protein functions. If proteins have a similar ancestral sequence (Lee et al., 2007), then their protein sequences are homologous. Given that proteins with similar sequences often perform similar functions (Blomberg et al., 1999), the recent use of convolutional neural networks (CNN) in genomics for sequence problems has ushered in the era of deep learning in computational biology. DeepBind and DeepSEA have been successfully used in modelling protein binding sequences with an efficiency superior to that of the best available conventional learning technologies (Zeng et al., 2016). In a previous study, we analyzed the findings by using the receiver operator characteristic and area under the roc curve score for enzyme protein data settings. Compared with the ECFP method for fingerprinting and graph convolution, our one-dimensional CNN approach was superior to SMILES representation (Kearnes et al., 2016). Numerous significant structures (i.e., motifs), such as protein sites, have been identified by one-dimensional CNN by using learning filters (Bengio et al., 2013). Important functional substructures (i.e., where a protein can bind) may also be omitted from “chemical motive”. Normal machine learning processes are limited by predefining features, by affecting prediction accuracy during the proper selection of functions, and by restricting model adjustments or modifications to flexibility (i.e., all preprocessing steps must be repeated). These drawbacks can be overcome by deep learning techniques that seamlessly extract inputs by using conventional gradient-based methods or by differentiating when their interpretability is required (Shrikumar et al., 2017). Shared data availability and ever-compute capacity outperform conventional approaches by profound deep learning methods, such as CNNs. By using convolutional filters, pooling, and completely linking layers, these deep learning methods imitate how the brain functions by leading the resulting network to concentrate on features that are essential to the resolution of controlled tasks. Several scholars have recognized the importance of proposing an approach for classifying transport protein category from their molecular functions on the basis of DDE profiles and biochemical properties, thereby providing a powerful prediction template. Le et al. (Ho, Q. T., et al., 2018, Le et al., 2019; Le and Nguyen, 2019) evaluated the classification performance of the Rab protein molecular functions and the identification of SNARE proteins. A deep CNN profile can obviously enhance various traditional methods for prediction measurement of protein functions. However, all DDE information must be incorporated into deep CNN better to avoid missing important information. Enzyme is a special type of protein, and sequence alignment typically tests the similarities among amino acid sequences. Protein prediction computing methods can be employed to fill the gap between sequence data and the unfamiliar characteristics of these proteins. The prediction of protein functions is generally considered an issue of multilabel classification. Researchers have tried numerous methods to address this problem (Wang, Z et al., 2011). As an example, a simple local alignment search tool (i.e., BLAST), which searches remote counterparts and uses the information of these homologous proteins to predict query sequence function, is the first widely used method for predicting protein functions. The most difficult method involves the direct use of protein sequences without any other resource for protein function prediction (Le et al., 2017). Several researchers are currently developing methods for enzyme classification. Jensen et al. (L. J. Jensen, et al., 2002) predicted the first classes of enzymes by using physical–chemical sequence-based characteristics and CNN (L. J. Jensen, et al., 2002). One-dimensional convolution is utilized to determine amino acid sequence-related features (Y. Li and Shibuya, 2015), whereas two-dimensional convolution is associated with position-specific scoring matrixes (M. Spencer, et al., 2014) or other function maps. The work of A. Ghulam et al. demonstrates (Ghualm et al., 2020) analysis a profound learning model based on a two-dimensional neural network (2D-CNN-PSPD) with prediction of a pathway protein domain. A. Ghulam et al. have suggested a model for composition (DPC) function extraction profile dipeptide deviation (DDE) model.

**FIGURE 4:**
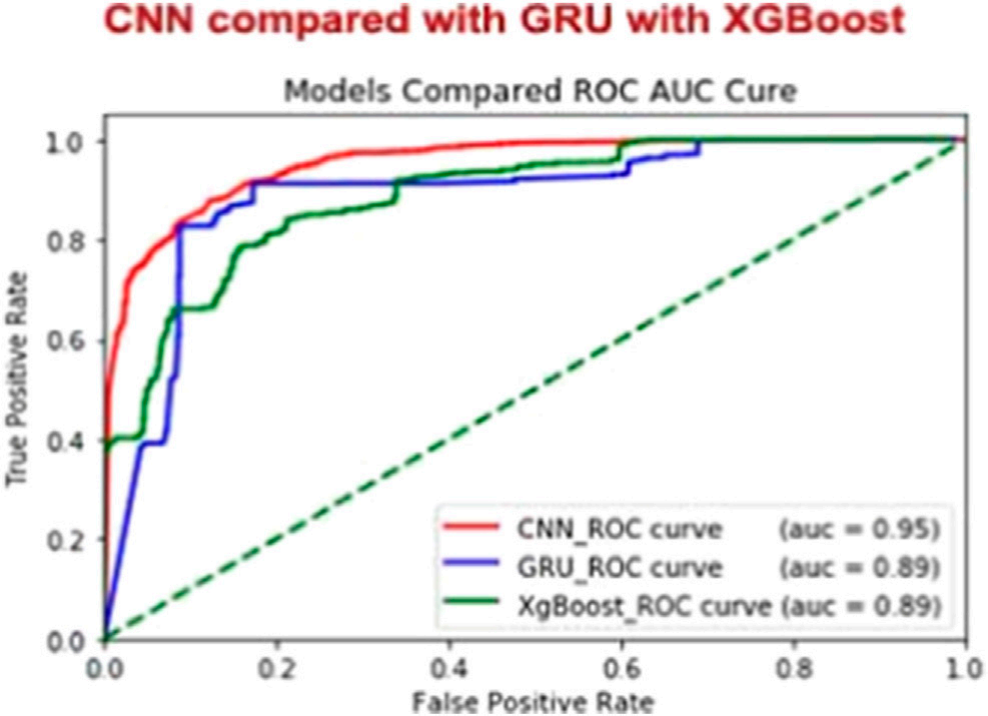
Comparison of CNN with GRU and XGBoost.

In this study, we proposed the use of a CNN and a classification level learning for predicting secondary protein structures. In protein sequence function prediction using deep learning techniques, especially CNNs, are used because they automatically utilize features after the data are translated into a suitable image format. Although the model proposed herein was evaluated in predicting enzymatic activity, this method was applied in predicting enzyme-specific proteins (E. I. Zacharaki, 2017). In the following sections, the implementation of the system is described. Moreover, the representation of protein structures, the configuration of the CNN method, and the network output fusion mechanism are discussed. The assessment and its results are then discussed and debated. The protein enzymes discussed in this work were from the UniProt Bio-database (https://www.uniprot.org/UniProt). An NCBI Protein database (https://www.ncbi.nlm.nih.gov/protein) entry indicates both an enzymatic protein and a non-enzymatic protein. The collected data from each DDE vector are standardized with a 400-length profile. The DDE model shows how score matrix functions can be created by the 400 vectors of the original protein profiles. All values are then synchronized with the same amino value, followed by a sequence-length dividing the amino acid frequency. Finally, all practical values are scaled using several formulas. The vector profiles are obtained from the mean and standard deviations of 400. The efficiency of this approach is compared with that of normal DDE by using real and simulated data sets for controlling overfitting. Moreover, this proposed approach is compared with a two-dimensional CNN (2DCNN), an approach that treats the DDE as images with vector profiles. The proposed CNN is then trained to enhance the predictive efficiency of our model in reducing overfitting. The optimization is based on the implementation of a stochastic gradient reduction. Network outputs are used as class probabilities following SoftMax standardization at the test stage. The Rectified Linear Unit (ReLU) activation function is also launched to incorporate nonlinearity to reflect the results well. The data are split into 5 -fold cross-validation. The data are tested separately and then cross-validated. The experiment is conducted according to a running model with age 80 to identify the best model with considerable results. A comparison of our proposed model with two other machine learning classifications, including GRU, reveals that our findings are consistent with those of a previous research that implemented XGBOOST. The performance of our proposed approach is considerably better than that of the other models (Figure 4). In summary, the results of this study demonstrate the precision, sensitivity, precision, and precision of MCC and ROC (AUC) of the proposed method in predicting secondary protein structures. The accuracy of this method on the uniform data set accuracy of the training set is 0.8762%. For non-normalized data collection, the test accuracy is 97.3. The training accuracy is 98.72. The proposed model is built into an independent data set via fivefold cross-validation.

## Materials and Methods

### CNN Proposed Method

**FIGURE 1:**
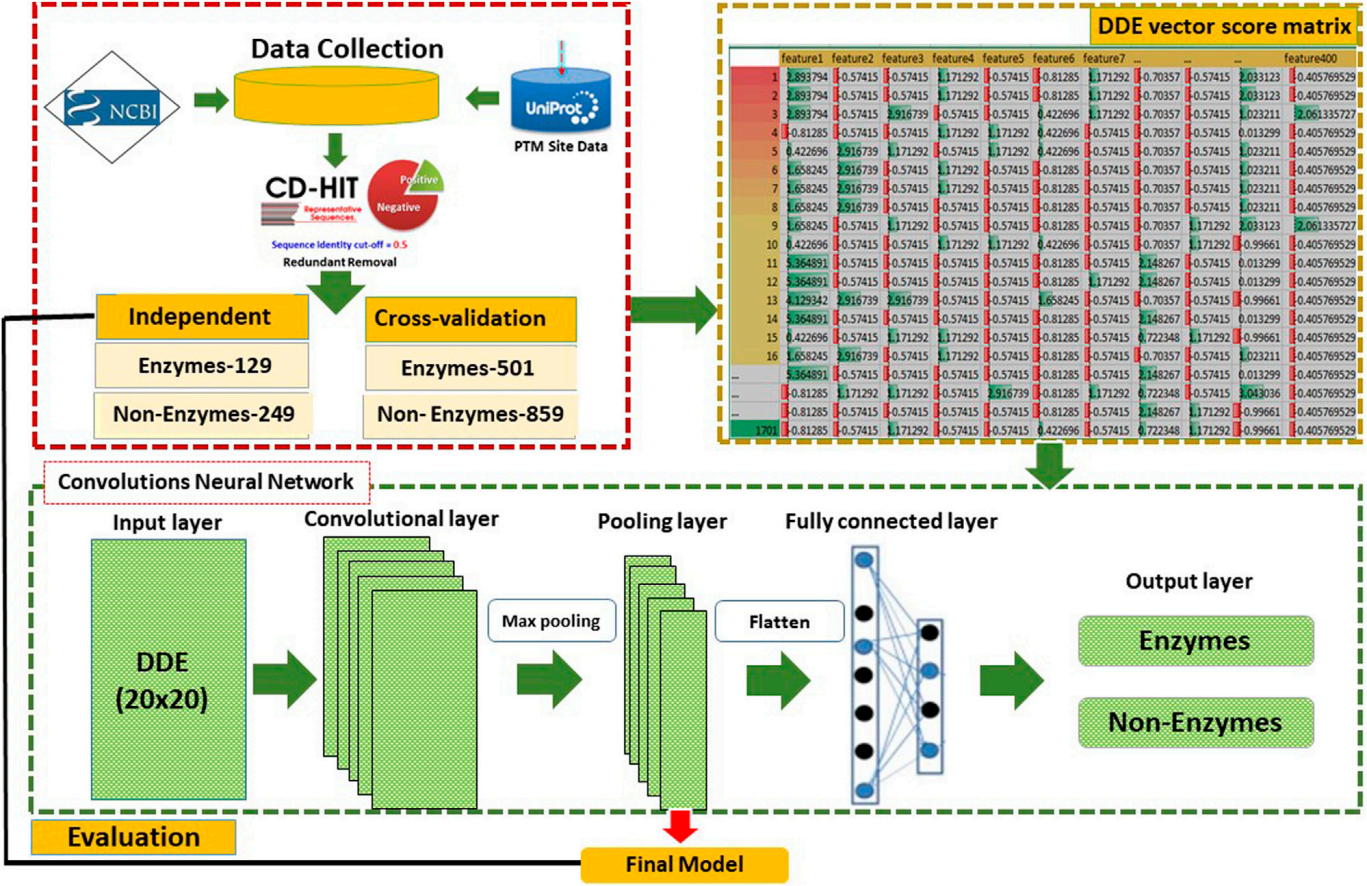
Proposed framework model.

We use new techniques to summarize the extracted features of proteins. The features are 400 vectors score matrix of the same amino acids that include the Convolutional neural network with DDE. This method has been effectively applied by several bioinformatics researchers. A number of scholars have proposed theories to explain conduct experiments on some of the commonly used machine learning algorithms, such as k-NN (Keller et al., 1985), Random Forests (Breiman 2001), and support vector machine (Chang and Lin, 2011; Cheng et al., 2019) but we have used GRU and XGBOOST classifiers for comparison results. Other comparing the proposed method with 2DCNN might seem counterintuitive because 2DCNN is an approach that treats DDE vector score matrix as images with vector profiles. The new techniques for using DDE vector profiles are used to summarize the same amino acids to generate a 20 × 20-dimentional vector and a neural convolutive 2D network. We implemented the DDE vector profiles method with a different vector score matrix and then set the cross-validation datasets as training datasets. An independent dataset means the data test is used as an input within 2DCNN. Our proposed method identified high-performance enzyme and non-enzyme proteins. The flow diagram and analysis of the study are presented in Figure 1. It includes various phases starting with data collection, preprocessing, and then feature extraction set development. Most experiments are performed via a 2DCNN method. A flow chart of the analysis is shown in Figure 1, and the details of the proposed method are defined as follows.

### Learning and Classification Using CNN Network

The proposed computational methods are based on 2DCNN learning and classification to resolve most problems in deep learning, machine learning, and data mining (Gao et al., 2019). Several algorithms are used to make a computer know what is required from 2DCNN experiment tools. Our proposed deep learning architecture includes a 2DCNN for extracting features to identify enzymatic proteins (Le et al., 2019). This deep learning architecture is a deep architecture of neural networks that can efficiently deal with sequential data in various fields (E. I. Zacharaki, 2017). Only the hidden layer of the essential gap can be seen between these two networks. A CNN method has several hidden layers from which deep characteristics and secret patterns can be identified. Herein, many hidden layers are built in a deep network to be fair for our research question.

### Convolutional Neural Network Structure

This study was conducted using 2DCNN (Torrisi et al., 2019; Cheng et al., 2019). CNN is successfully implemented in various applications. Figure 1 reveals that 2DCNN is comprised of 2D layers. The input layer parameters are included in the DDE vector profiles of 20 × 20matrices. The transport of enzymatic proteins is identified by using their DDE vector profiles as features vectors. We then trained our CNN to improve our model’s predictive efficiency and control overfitting. We used input as image of the window size by 20 × 20 length vectors profile features that result from the data. We assumed that in the layers to implement CNN, the DDE vector profiles of the 20 × 20matrices look like an image pattern. Consequently, the input in the CNN model (M.S. Klausen, et al., 2019; Jones, 1999; Jones and Kandathil, 2018) is like DDE vector profiles or an image pattern. More hidden layers can quickly be used to distinguish non-enzyme and enzyme proteins in the CNN network. The combined filter layers (with 32, 64, and 128 filters) and additional parameters in this analysis are listed in Table 1.

**TABLE 1:** Parameters of the CNN model settings.

Parameter name	Recommendation
Learning rate	0.001
Activation function	ReLU
Cost function	Cross-entropy
Optimizer	Adam
Dropout rate	0.4
Width	3
Depth	128
Epoch	80

For three consecutive convolutional computational blocks, batch normalization, and rectified linear unit activation (Nair and Hinton, 2010), optional (the dropout ratio is set) and max-pooling layers are used in the architecture proposed by the CNN system to completely connect the layers (Qi et al., 2018). These effects are less shocking if local features are taken into account; the co-evolutionary layer tests the neuronal output related to local input regions. A 2D convolution between a series of filters is added to each input channel. By summing the results across every channel, the 2D activation maps are calculated, and then each filter is stacked to generate a 3D output volume. Batch standardization normalizes every function map channel by spatial locations and batch instances by averaging. In ReLU, the activation function, as with the max (0, x) threshold, is an element-sensitive activation. The drop-out layer is used by chance to lower the overfitting of CNN devices during preparation. The dropout is evaluated by conducting content analyses to validate and separate sets for the set purpose. The bundling layer conducts a down-sampling procedure in the spatial dimensions to capture the key global elements with a fixed length. The last layer is totally connected, and the class values are predicted.

Normalizes every function map channel by spatial locations and batch instances by averaging. In ReLU, the activation function, as with the max (0, x) threshold, is an element-sensitive activation. The drop-out layer is used by chance to lower the overfitting of CNN devices during preparation. The dropout is evaluated by conducting content analyses to validate and separate sets for the set purpose. The bundling layer conducts a down-sampling procedure in the spatial dimensions to capture the key global elements with a fixed length. The last layer is totally connected, and the class values are predicted.

### Training Cross-Validation and Independent as Test Sets

All analyses are conducted using the expected CNN output as a probability vector. Each enzyme group identifies mechanisms through which the CNN’s output is determined by employing a loss feature that imposes a penalty on classification errors. The CNN parameters are trained to minimize this average loss over the noted (workout) samples. The SoftMax loss function (i.e., the SoftMax operator followed by the logistic loss function) is used to evaluate probability distribution across groups. Optimization is based on a stochastic gradient downgrade implementation. At the test point, network outputs are used as class probabilities following SoftMax standardization.

### Multiple Layers Generating for Deep Neural Network

This study is conducted using CNN, which is the largest deep neural network. CNN is effectively used in many fields, in computer vision, especially where the input typically is a 2D pixel image density matrix (Lakhani and Sundaram, 2017). Herein, the 2D structure of the CNN input image architecture (Krizhevsky et al., 2012; Yasaka et al., 2018) is used, and the equivalent 2DDE vector–matrix inputs with a dimension of 20 × 20 are conveniently manufactured. The model 2DCNN aims to accumulate the hidden figures in the profiles of the vector m instead of 2D. The DDE feature profiles are then connected from the input layer to the output layer through a few hidden layers to the 2DCNN architecture. Figure 1 explains the mechanism by which a DDE-vector profile is embedded in a CNN model and then transferred through a series of convolutive, nonlinear, down sampled, and completely connected layers and, eventually, outputs.

The traditional CNN architecture (Wellem, et al., 2018) is typically adopted to develop the deep enzyme architecture we have designed in CNN. Seven secret layers, including a 2D convolution layer, a single activation feature, a pooling layer, a flattening layer, and completely connected layers are used in our model. The first layer in a CNN is an overlapping layer. In particular, a DDE profile in the first layer of our CNN is a method for performing 2D convolution, with some existing parameters, such as *nxn* kernel size, f filters, *1X1* steps and *1X1* zero padding. The respective motif features were filtered through convolutional operations. We also introduce an activation function for ReLU to integrate non-linearity so that the model can better reflect our performance. Additional 2DCNN max-pooling layers are added to minimize matrix size and remove non-maximum values and overfit power, including *1X1* additional path measurements. We add flattening layers to flatten the input before applying the fully connected layers. The sigmoid activation function is used following this step to determine whether or not every neuron can be activated.

CNN method consists of several layers with various parameters and output types. They are composed of 2D zero padded layers, 2D converting layers, and 2D max packing layers and filters with different numbers. To construct a high-quality model, we specify several layers with various parameters in the hidden layer. Figure 1 provides an explanation for 2D converting layers, 2D max packing layers, and 2D zero padding layer, as well as complete 2D pooling layer. For these layers and parameters, the following can be specified.

#### Zero Padding 2D Layer

In zero padding layer, we set and add the values at the beginning and end of the 20 20 matrices. The zero padding layers allow us to apply the filter to the border of the output values. The matrix is shifted to a dimension of 20 20, and zero padding is applied to this matrix. Zero padding is a mechanism that enables one to maintain the original size of the input. With each convolutional layer, just as we determine how many filters to have and the size of the filters, we may also decide whether or not to use the padding.

#### Convolution 2D Layer

In 2D matrix convolution, 3 × 3 sizes are added. We have studied the kernel sliding windows with tiny 3 × 3 matrices and turn to other matrices until the end. The phase of 2D convolutional layers is shown in Figure 1. It has a dense layer, a dense output layer, and three convolutional layers. The input size of the images coming into this CNN is defined as 20 × 20, and the filter size of the first convolutional layer is 3 × 3, which is defined in Keras with parameter kernel size. The 5 × 5 size is defined by the second conv layer, whereas the third conv layer is defined by 7 × 7.

#### 2D Layers of Max Pooling

Usually, these layers stand after convolution layers. The maximum pool layers have several parameters, i.e., loop size and stride. In this study, we use two measures to build the 2D layers of max pooling. The goal of using max pooling layers is to delete all maximum values in that filter and to reduce time consumption in the next layer.

#### Flatten Layers

Layers are applied to flatten the data to turn the data matrix into a vector. Application layers are also used in the output layers. Between the convolutional layer and the fully connected layer is a “flat” layer. Flattening transforms a 2D feature matrix into a vector that can be fed into a fully connected neural network classifier.

#### Dense Layers

The layer is validated using a regular, fully connected neural network. Provided that all nodes in the previous layer are interconnected, nodes with completely connected networks can quickly learn from the previous nodes. Therefore, our model will learn a lot of data and perform better. Using sequential (dense) layers, one or more completely connected layers can be added. With a dropout layer, we can usually follow each fully linked layer and learn more about dropout in our Neural Network Hyperparameter Guide by using sequential (dropout) layers.

#### Dropout Layers

Dropout layers are added to improve our model’s predictive efficiency and avoid overfitting. In this work, the drop-off values are integer values between 0 and 1. Dropout layers are used to mask portions of their output through random means to avoid overfitting (Srivastava, A., et al., 2014). This occurs when the remaining neurons are multiplied by 1/p (where p represents the likelihood of an element being dropped) and by removing the random part of hidden neurons. For our implementation, the dropout ratio is set at 0.4.

#### Rectified Linear Unit (ReLU)

ReLU is the activation function used to discriminate against enzyme proteins during CNN implementation. For all deep neural networks, ReLU is the primary activation function. The characteristic of ReLU activation is defined as:

f(x)=max(0,x) (1)

where x is the number of neural network inputs.

#### Softmax

Several aspects of this study warrant further discussion. Some of these aspects are the use of SoftMax activation function to examine circumstances under which the final output layer consists of two neurons corresponding to the two classification effects. The two neurons are fully bound to the previous layer. Tensorflow (Abadi, 2016) and TF. Learn (Tang, 2016). Overall, these studies support the validity of implementing the deep learning CNN architecture. SoftMax (normalized exponential function) is composed of linked layers that are a formula-defined logistic function:

$$\sigma {\left(z\right)}_{i}=\frac{e^{z_{i}}}{\sum_{k=1}^{k}e^{z_{i}}},$$ (2)

where *z* is the *K*-dimensional vector input vector, the *K*-dimensional vector *r(z)* is the actual range (*0, 1*) values, and the *j*th class is the expected likelihood of the sample vector of *x*.

A possible interpretation of this finding is that various layers and the overall number of trainable 147,682 parameters found in 2DCNN are shown in Table 2. At present we determine the same number of layers and the majority of the parameters in each layer across all five classification challenges. There was only one non-shared parameter among the five classes, and that was the dropout value. As such, each of them has a separate optimal dropout value. So, five complexes categorization can produce a dropout value of 0.2, 0.1, 0.1, 0.2, and 0.1. Our model is consistent with all complexes of the electron transport chain due to this.

**TABLE 2:** Used all model layers and trainable parameters in 2DCNN.

Layer (type)	Output shape	Param #
conv2d_4 (Conv2D)	(None, 1, 20, 32)	5,792
leaky_re_lu_5 (LeakyReLU)	(None, 1, 20, 32)	0
max_pooling2d_4 (MaxPooling2	(None, 1, 10, 32)	0
dropout_5 (Dropout)	(None, 1, 10, 32)	0
conv2d_5 (Conv2D)	(None, 1, 10, 64)	18,496
leaky_re_lu_6 (LeakyReLU)	(None, 1, 10, 64)	0
max_pooling2d_5 (MaxPooling2	(None, 1, 5, 64)	0
dropout_6 (Dropout)	(None, 1, 5, 64)	0
conv2d_6 (Conv2D)	(None, 1, 5, 128)	73,856
leaky_re_lu_7 (LeakyReLU)	(None, 1, 5, 128)	0
max_pooling2d_6 (MaxPooling2	(None, 1, 3, 128)	0
dropout_7 (Dropout)	(None, 1, 3, 128)	0
flatten_2 (Flatten)	(None, 384)	0
dense_3 (Dense)	(None, 128)	49,280
leaky_re_lu_8 (LeakyReLU)	(None, 128)	0
dropout_8 (Dropout)	(None, 128)	0
dense_4(Dense)	(None, 2)	258

### Data Collection

The protein enzyme entries of the UniProt Bio-database (https://www.uniprot.org/uniprot) in this paper are evaluated. The collection of enzymatic protein and not enzymatic proteins are in taken from NCBI Protein database (https://www.ncbi.nlm.nih.gov/protein) entrance. First, data are collected from the UniProt database, a detailed tool for detecting enzyme protein or non-enzyme protein (Poux et al., 2014). The entire sequence with the annotation “protein level proofs” is selected. Subsequently, sequences of the similarities are deleted to prevent overfitting the dataset problem. The collected sequences are from the data sets for preprocessing data: cross-validation and independent data sets. Table 3 lists all data sets with cross-validation and independent datasets used in this study.

**TABLE 3:** Statistics of all retrieved Enzyme Protein and Non-enzyme proteins.

	Used data points	Training	Independent
Enzyme protein	652	501	129
Non-enzyme proteins	1,108	859	249

### Dipeptide Deviation Extraction Mean (DDE Models)

The data of every DDE vector feature profile with a size of 400 are normalized. The DDE model shows how the 400 vector features score matrix functions from the original protein profiles can be generated. All values are first synchronized with the same amino value. The amino acid frequency is then divided by sequence length. Finally, all functional values are scaled using the following formula: X. Means and standard deviations generated from 400 vector features are obtained from vector score feature profiles. All vector profiles are configured into the DDE and then 2D method. Dipeptide composition (Bhasin and Raghava, 2004) has been used in predicting different types of protein sequence functions (Dhanda et al., 2013).

Therefore, we have also created a novel amino acid composition-based function descriptor, namely, Dipeptide Deviation from Expected Mean (DDE), for the effective separation of enzymes and non-enzymes from protein sequence structures (Saravanan and Gautham, 2015). The vectors of the function differentiate the distance between a real sequence and a representation of a binomial and uniform distribution theoretical sequence. DDE does not focus on alignment of protein relationships but enables end-users to imagine interrelationships within a set of proteins without having a prior assumption of those proteins (Carr et al., 2010). Previous studies (Chen et al., 2014; Saxena et al., 2018) reported differences in the composition of dipeptides between enzymes and non-enzymes. We use the composition aspect of dipeptides to calculate the variance of dipeptide frequencies from expected mean values. Concerning the variance from the predicted mean, we call this behavior DDE. Three parameters were determined to generate the DDE function vector: Scale of the composition of dipeptide (Dc), mean theoretical value (Tm), and variance theoretical value (Tv).

These three parameters and DDE are determined. DC 1) is the dipeptide composition calculation for dipeptide I in peptide P:

$$D_{c\left(i\right)}=\frac{n_{i}}{N}.$$ (3)

A total of 400 possibilities (20 × 20 regular amino acids) are available for dipeptides. However, not all of them will happen in all sequences. Dipeptide I exists at frequency N, and N is at l-1 (i.e., in P, the number of potential dipeptides). TM 1) indicates the theoretical average:

$$T_{M\left(i\right)}=\frac{C_{i1}}{C_{N}}\times \frac{C_{i2}}{C_{N}}$$ (4)

where C_*i*_1 is the number of the first amino acid codons, and C_*i*_2 is the number of the second amino acid codons in the specified dipeptide i. The total number of codons possible is the CN minus three stop codons (i.e., 61). Given that *TM* (i) measurement is not based on peptide P, all 400 dipeptides are pre-computed at once by the *TM*[i] method. *TV* (i) provides the theoretical variation of dipeptide I:

$$T_{v(i)}=\frac{T_{M(i)}(1-T_{M(i)})}{N}.$$ (5)

The theoretical mean of *I* as determined using *Eq.* is *TM(i)* 2, and *N* is again *l-1*, the *P* number of dipeptides. Finally, it calculates *DDE(i)* as

$$DDE_{\left(t\right)}=\frac{D_{c\left(i\right)}-T_{m\left(i\right)}}{\sqrt{T_{V\left(i\right)}}}.$$ (6)

For each of the 400 dipeptides, DDE is computed. The 400-dimensional function vector is

$$DDE_{p}=\left\{DDE_{\left(i\right),}..._{,}...DDE_{\left(n\right)}\right\},where,i=1,2,...,400.$$ (7)

## Evaluation and Performance

The data presented herein provide evidence the superior predictive test performance of our proposed method in terms of accuracy, sensitivity, precision, MCC, and ROC (AUC) (i.e., the coefficient of similarity of Matthew). In our definitions, TP, FP, TN, and FN stand for true positive, false positive, true negative, and false negative. The threshold is based on sensitivity, characteristics, precision, and MCC, and the threshold is chosen to maximize the balance between sensitivity and specificity.

$$Sensitivity\;=\;\frac{TP}{TP+FN}$$ (8)

$$Specificity\;=\;\frac{TN}{TN+FN}$$ (9)

$$\mathrm{Accuracy}=\;\frac{TN+TN}{TP+FN+TN+FN}$$ (10)

$$\mathrm{MCC}=\;\frac{TP\times TN-FP+FN}{\sqrt{\left(TP+FP\right)\left(TP+FN\right)\left(TN+FP\right)\left(TN+FN\right)}}$$ (11)

## Results

### Assessment of Predictive Ability

**FIGURE 2:**
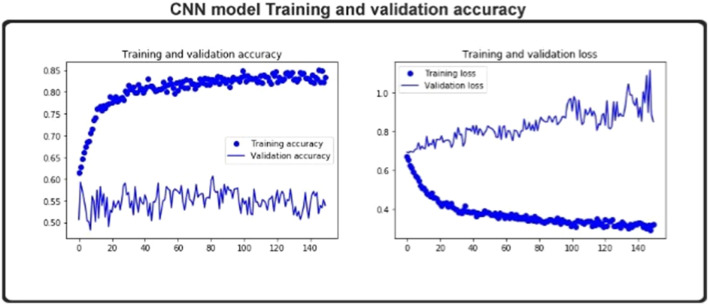
Training and validation accuracy and loss predicted score.

One iteration of each batch of cross-validation data updates the model parameters during the training process and, at this stage, will reliably achieve training cross-validation. Only the independent collection is used to assess test accuracy at the end of each training cycle without intervening in model parameters. As shown in Figure 2, the training’s precision steadily converged with the number of training periods. Therefore, model training is adequate for 150 epochs. The detailed testing of the model’s generalization skill is always less than the precision of the teaching.

In Figure 2, the blue line bubbles indicates improvements in the iteration times and the accuracy of the training. The blue line depicts the number of iterations and the improvement in the accuracy of the experiment’s cross-validation in preparation. Only after the data are educated can the corresponding parameters be calculated. The precision of the test set is then obtained. During the workout, the iteration process influences the parameter description, which in turn influences the results of the test. The difference in prediction precision is seen in the figure with the number of iterations. A loss function, which assigns a penalty to classification errors, may be used to calculate the model output. The test loss value for each cycle is shown in Figure 2.

We test the model on the structures of both normalized and non-normalized data. The structured information includes equivalent numbers of individual class instances. Cross-validation data sets have a 400 length DDE vector and separate datasets that include 400 length features on the same data points. Test precision on the uniform data set is 0.85.6, and training exactness is 98.72. Test accuracy is 97.3 for a non-normalized data set. The precision of the training is 98.72. The difference between the two is minimal. We then plot the training and testing learning curves of the model to obtain more details.

Figure 2 demonstrates the model’s training accuracy on the structured data and in checking individual learning sets. The normalized learning curve indicates that it has reached the maximum precision in approximately 150 epochs. Although the accuracy of the non- normalized learning curve took several years, it did not attain the accuracy of the normalized learning curve. We have selected 1,360 enzymes from the PDB database, excluding enzymes that generate several responses and are linked to many enzymatic functions. The number of samples per class is listed in Table 1. The data set has been divided into five folds.

For the training set, four folds and one test set are used. For preparations and validations, three folds of the training set are used. Cross-validation is performed to change the model parameters. The test output is calculated after selecting the model parameters. We compare the proposed model with the architecture two of Evangelia in this experiment. The same and training sets are used in both approaches. The median values are slightly different (Table 4) because the number of search samples and comparative experiments are required. The red figures in Table 4 show the proportion of each enzyme successfully predicted; these figures are the true positive rate. The accuracy of Evangelia and SRN is 90.83 and 92.08%, respectively.

**TABLE 4:** CNN identification of the optimal parameter for various models.

	Cross-validation				Independent			
Model	ACC	Sensi	Speci	MCC	ACC	Sensi	Spec	MCC
DDE-CNN	0.8762	0.9028	0.8497	0.7545	0.7621	0.7621	0.7621	0.5276

### CNN Model Predictive Ability of ROC (AUC) Curves

In predicting enzyme protein sequences, we define both the positive and negative data for non-enzyme proteins. The data set and training data set are all independent proteins of this kind. We performed fivefold cross-validations to build our model with considerable success into our independent data set. We split the data into 5-fold sections of the same data set, followed by the independent data set as the test data. The other cross-validation datasets are used as the training data. The original dataset is reused to test the independent experimental data set. The identification performance of our proposed model is shown in Table 3.

In the enzyme protein sequence, we defined positive data and negative data for non-enzyme proteins. The data set and training dataset are all independent proteins of this kind. We used five-fold cross-validations to build our model with considerable success into our independent dataset. It implies we have split data into ten 5-fold sections of the same, followed by independent as test data, and then the other cross -validation datasets as training data. To order to test the experimental independent dataset, the original dataset was reused our proposed model identification performance as shown in table 4.

**FIGURE 3:**
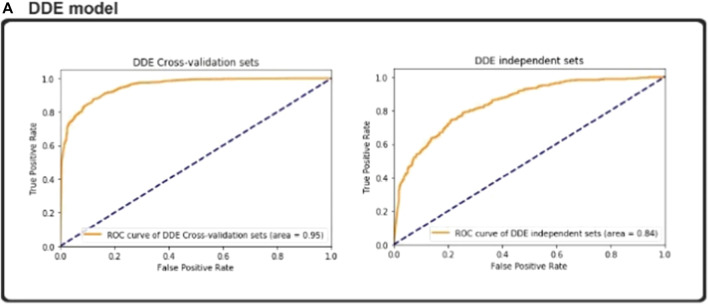
CNN model ROC (AUC), precision and recall.

The ideal subset of features is considered as class labels, which are inserted as variables in the CNN classifier, and the independent test data sets are expected to have enzymatic proteins. AUC, ACC, Sn, Sp, and MCC values are determined via the fivefold cross-validation. The ROC curve and precision–recall are drawn to determine the model’s predictive efficiency (Figure 3).

### Improvement in the Performance and Prevention of Overfitting in Identifying Enzyme and Non-enzyme Proteins

The amount of time used in the experiment substantially influences the performance result. Our findings are consistent with previous results showing the best time from the first to the 150 epoch. We encourage further research examining the best model with considerable performance to conduct our experiment following a running model with 150 epochs. In this section, we used different parameter then achieved the results of the analysis summarized in Table 5. When attempting to change the dropout value from 0 to 1. As shown in table. The aim is to improve the performance of the neural network and ensure overfitting within the data sets. We can see that the results improved and compared to the other performers and reached Sens 0.899, Spec 0.876, Acc 0.888, and MCC 0.778 based on cross-validation. The respective Sens 0.799, Spec 0.810, Acc 0.805, Mcc 0.614 based using test independent sets. These output values are 80.5%, 61.4%, respectively based on MCC, in separate datasets. Hence, this dropout value is used to build our final model.

**TABLE 5:** Comparison with different filters.

Filter cross-validation					Independent			
	Sens	Spec	Acc	MCC	Sens	Spec	Acc	Mcc
32	0.899	0.876	0.888	0.778	0.799	0.810	0.805	0.614
32–64	0.895	0.858	0.877	0.756	0.763	0.828	0.795	0.596
32–64–128	0.894	0.862	0.878	0.759	0.793	0.855	0.824	0.657

### Comparison of the Proposed System With Other Classifiers

These findings are less surprising because we have used the same data set and compared the other major classifiers with our method. A possible reason for this discrepancy might be that we have used the results from both cross-validation and independent data sets and the comparison. We have used GRU (Baccouche et al., 2020) and XGBOOST (Pang et al., 2019). The performance at the same stage of the proposed method and the other classifiers is summarized in Table 6. Results show that both the cross-validation and independent data sets perform better than the other methods. Several findings of this comparison, such as the results of cross-validation and independent data sets, must be discussed in detail. Our method predictions results are consistent with previous results showing ROC AUC based on comparison of other two machine learning classifiers, such as GRU (Baccouche et al., 2020) and XGBOOST XGBOOST (Pang et al., 2019). According to the results of our comparison, the performance of our proposed method is considerably better than that of other methods (Figure 4).

**TABLE 6:** Efficiency of different methods in terms of various feature extraction schemes.

	Cross-validation				Independent			
Classifiers	ACC	Sensi	Speci	MCC	ACC	Sensi	Spec	MCC
CNN	0.8762	0.9028	0.8497	0.7545	0.7621	0.7621	0.7621	0.5276
GRU	0.8584	0.8757	0.8411	0.7481	0.9001	0.9459	0.8540	0.8034
XGBOOST	0.8088	0.7530	0.8646	0.6445	0.9055	0.8111	1	0.8242

### ROC (AUC Curves With Various Dimensional Reduction Methods

DDE features extraction is often used for experiments that involve different experiment data sets. We encode the features of enzymatic proteins from the fasta format by using the vectors matrix with the DDE function with a length of 400. We then use different data sets to implement the DDE process experiment. After removing redundant and unnecessary information, we obtain vector matrix features with a length of 400. Optimum parameters are calculated in DDE, after then we used a DDE function to with selected features. We then introduce the CNN model classification for the predicting the functions of enzymatic protein sequences.

### Hyperparameter Optimizer Performance

**FIGURE 5:**
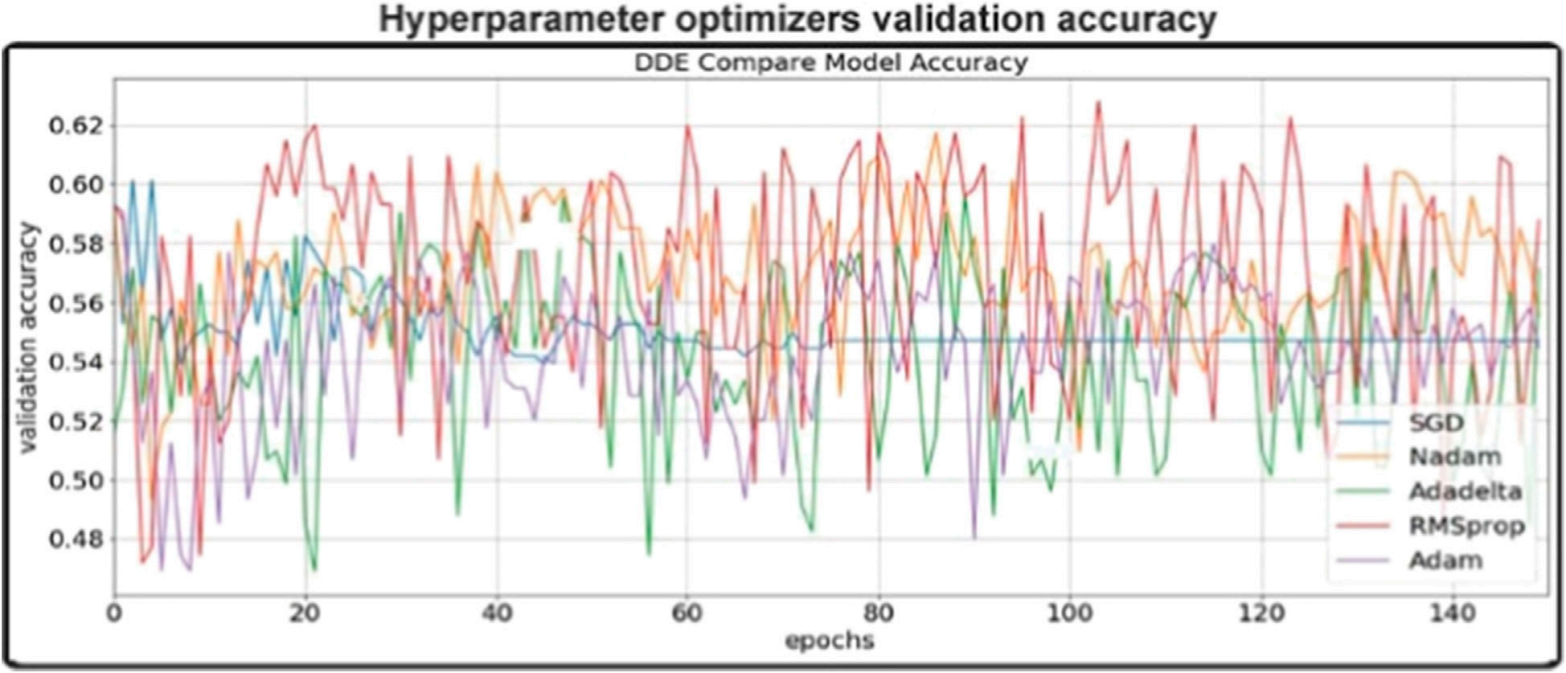
validation accuracy of different optimizers in this study (The epoch ranges from 0 to 150).

The hyperparameters used differ from parameters of a model trained in architectural context propagation. The choice of such hyperparameters depends on a number of factors if a profound learning model is created. The performance of the model is greatly affected. For example: the number of overlapping layers, the number of filters in each layer, the number of epochs, the dropout rate and the optimizers, which affect the profound learning model. We need to define a number of parameters to speed up the workout and avoid duplication in order to change hyperparameters. As Chollet (F.Chollet, 2015) has suggested, every step of the above mentioned method for tuning hyperparameters is incorporated as follows into the process of tuning:

Different optimizers: Rmsprop, Adam, Nadam, Sgd and Adadelta optimized the validity precision of different optimizers in this trial dependent on CNN networks. For each optimization phase, the prototype was reset, i.e. a new network was created to allow the different optimizers to be comparable equally. In general, the results are illustrated in Figure 5 And we wanted to build our final model for Adam, an optimizer with consistent results. In the same field, Adam is also a preferred optimizer (N.Q.K. Le et al., 2018). As per Figure 5. We have found that our validation accuracy compared to the training accuracy did not improve after the 150th epoch. Therefore, at the 150th level, we agreed to complete our preparation to cut down on training time and to avoid overfitting. Then we tuned for the best outcomes of this data set the other hyperparameters within our model (e.g. learning rate, batch rate or drop-out rate).

### Confusion Matrices Predicted Lables

**FIGURE 6:**
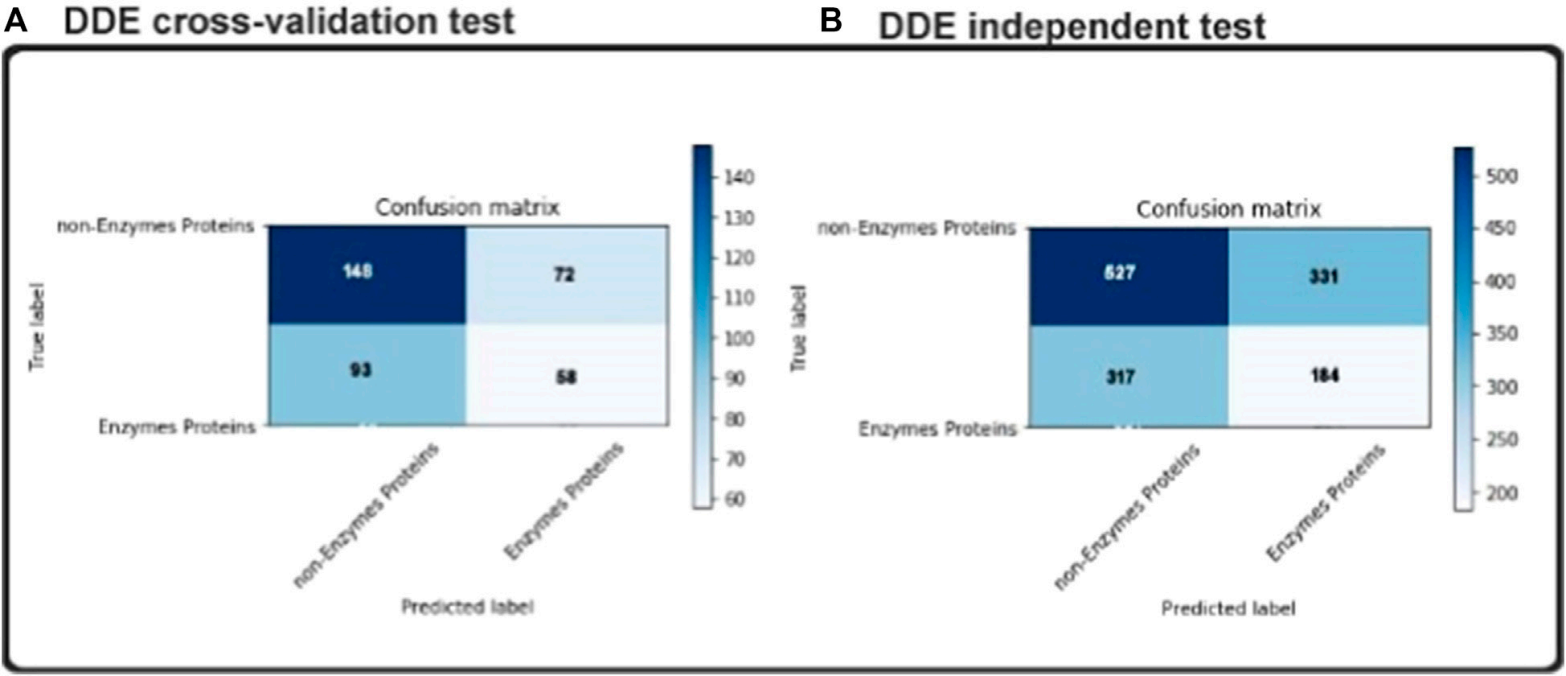
Confusion matrices of: **(A)** independent test **(B)** cross-validation test.

Overfitting is the big challenge in all matters of machine learning that our classification will only work well in our training sets, only if an undefined dataset gets worse. We have therefore also used an autonomous test to make sure our concept fits also in a blind dataset. As noted in the previous section, 151 enzymes and 249 non-enzymes were in our independent dataset. None of the samples appeared in the exercise set. In the Fig two matrices of uncertainty are shown as informative results. As shown in Figure 6 and consistent with our independent test results’ cross-validation findings. To be more detailed, our model achieved accuracy 80.5% in independent datasets outcomes. Compared to the cross-validation result, there are not enough variations, and it should illustrate that there was not any overfitting in our model. The use of dropouts in our CNN structure and the overfitting was shown to be effective (N. Srivastava et al., 2014).

### Comparison With Previous Methods

Previous research (Zhang et al., 2020) has supported the hypothesis that used a feature selection approach to construct a support vector machine (SVM)-based predictor to classify human enzymes using the amino acid composition (AAC), the composition of -spaced amino acid pairs (CKSAAP) and chosen informative amino acid pairs. To train and test the proposed model, a training dataset with 1,117 human enzymes and 2099 non*-*enzymes was created, as well as a test dataset with 684 human enzymes and 1,270 non*-*enzymes. A protein-protein interaction (PPI) network-based approach for predicting enzyme families was created in this study. As an example, the jackknife test had a success rate of 62.86 percent in identifying enzyme family class (Niu et al., 2015). The positive results suggest that the predictor described in this work might be beneficial in enzyme research as shown in table 7. The approach was used to estimate the enzymes-specific protein domain association prediction and the accuracy was 0.8762% percent, which is a significant increase above the accuracy obtained in our earlier work 73.5% percent in (Amidi et al., 2016) when just structural information was used.

**TABLE 7:** Existing method comparison accuracy and ROC(AUC).

S.no	Previous method	Accuracy	ROC(AUC)	References
1	SVM classifier. jackknife cross-validated	76.46%	0.8019	Zhang et al. (2020)
2	Jackknife test in identifying	62.86%	0.7898	Niu et al. (2015)
3	Support Vector Machines (SVM) and NN	73.5%	—	Amidi et al. (2016)
4	Proposed 2DCNN with DDE	Cross-validation 0.8762%, Independent 0.7621%.%	0.95%	—

## Discussion

Enzymatic and Non-enzymatic protein sequence datasets are used as cross-validation and independent experiment datasets. By using the vector matrix with the DDE function with a length of 400, we encoded enzymatic protein features from the fasta format. We obtained vector matrix features with a length of 400 after removing redundancies and unnecessary materials. We used the proposed CNN model to show a superior model for the entire set of filters, such as 32, 64, and 128 parameters, by using a fivefold validation test set as independent classification. We conducted all analyses using these filters in our proposed CNN method, which achieved a cross-validation accuracy score of 88.8%. Independent validation accuracy was 80.5%. Additional variables were derived on the basis of ROC AUC with fivefold cross-validation ROC (AUC) of 0.95%. We compared the performance of our proposed model with that of other models. The sensitivity of our model was 0.9028%, specificity was 0.8497% metrics, and overall accuracy was 0.9133–0.8310% MCC score.

## Conclusion

We used the CNN method proposed herein to differentiate explicitly enzyme proteins and other protein functions. The use of CNN in computational biology, particularly in protein function prediction, may also improve our results. A structure-based analysis of protein function shows ties concealed at the sequence level and provides a framework for understanding biological complexities on a molecular basis. The novel and innovative approach proposed herein is for describing 3D structures as a “batch of atoms (amino acids)” with geometrical characteristics and for exploiting the derived feature maps encoded with the DDE. We show that a DDE features extraction model with a different vector score matrix choice is generally more effective in reducing the chance of overfitting. Given the prediction for enzymatic activities is measured, the tool is the proteins based on the enzyme. The profound information model used to predict protein functions can provide rapid notes on extensive datasets, which opens up opportunities for applications, such as pharmacological target identification. However, there may still not be enough data available to accurately analyze the efficiencies and the confusing factors of evolutions in creating the system of non-homologous proteins. This system promises to accurately predict the functions of structural proteins.

## Data Availability

The data sets analyzed are open to the public through this study. The complete datasets can be found in the NCBI protein database for enzymatic protein. Then, in the data availability directory, we have applied all our data preprocessing to our fasta protein data collection and then can be removed similarity from the CD HIT web tool (http://weizhong-lab.ucsd. Furthermore, on request from the respective author, the code used to help the studying findings is available. The protein enzyme entries of the UniProt Bio-database (https://www.uniprot.org/uniprot?) in this paper are evaluated. The collection of enzymatic protein and not enzymatic proteins are in taken from NCBI Protein database (https://www.ncbi.nlm.nih.gov/protein) entrance. First, data are collected from the UniProt database, a detailed tool for detecting enzyme protein or non-enzyme protein. Further inquiries can be directed to the corresponding author.
